# Beyond glycemic control: molecular mechanisms of metformin in modulating cytokine networks in polycystic ovary syndrome

**DOI:** 10.3389/fendo.2026.1749906

**Published:** 2026-02-09

**Authors:** Amin Ullah, Yongxiu Chen, Fan Zhang, Bairong Shen

**Affiliations:** 1 Department of Endocrinology and Metabolism, Institutes for Systems Genetics, Frontiers Science Center for Disease-related Molecular Network, West China Hospital, Sichuan University, Chengdu, Sichuan, China; 2 Gynecology Department, Guangdong Women and Children Hospital, Guangzhou, China; 3 Health Management Center, General Practice Medical Center, West China Hospital, Sichuan University, Chengdu, Sichuan, China

**Keywords:** inflammation, metformin, molecular mechanisms, polycystic ovary syndrome, pro and anti-inflammatory cytokines

## Abstract

Polycystic ovary syndrome (PCOS) is a well-known hormonal and metabolic condition linked to immune system irregularities and persistent inflammatory responses. Cytokines play a central role in PCOS, contributing to insulin resistance (IR), ovarian dysfunction, and systemic inflammation. Metformin (Met), a first-line treatment for IR, exhibits immunomodulatory properties beyond its glucose-lowering effects. This review critically evaluates the molecular mechanisms by which Met modulates pro- and anti-inflammatory cytokines in PCOS, synthesizing preclinical and clinical evidence while highlighting inconsistencies and therapeutic implications. Met suppresses inflammation by reducing pro-inflammatory cytokines such as IL-6, IL-1, IL-17, TNF-α, and others. Met also regulates TGF-β signaling, mitigating ovarian fibrosis while promoting follicular development and oocyte maturation through increased expression of TGF-β family members such as GDF-9 and BMP-15. These effects highlight Met’s dual role in modulating inflammation and fibrosis. Additionally, Met influences inflammatory chemokines such as CXCL13, fractalkine, and others, further regulating immune responses and reducing inflammation. Moreover, combining Met with anti-inflammatory agents, such as resveratrol and probiotics, shows synergistic benefits in PCOS management. Understanding Met’s immunomodulatory mechanisms offers new insights into its therapeutic potential beyond glucose metabolism. Future large-scale, phenotype-stratified clinical trials are warranted to validate these mechanisms and translate the immunomodulatory potential of metformin into tailored therapeutic strategies for PCOS.

## Graphical Abstract

**Figure:**
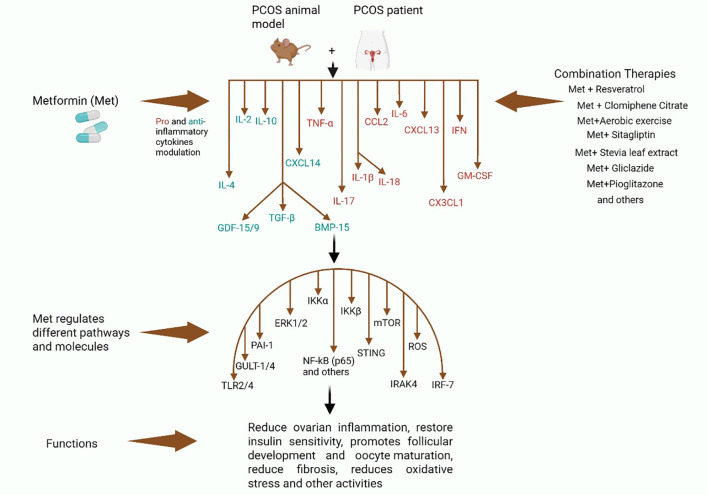

## 1 Introduction

PCOS is a heterogeneous endocrine and metabolic disorder characterized by chronic low-grade inflammation, hyperandrogenism, and ovarian dysfunction, affecting 5–20% of women of reproductive age worldwide (1, 2). While its etiology remains multifactorial and incompletely understood (3), immune dysregulation and cytokine imbalance are increasingly recognized as central to its pathogenesis. It is characterized by globally recognized symptoms, which include ovarian dysfunction, androgen excess, and polycystic ovaries (4). In women, hirsutism, acne, and alopecia are prevalent clinical manifestations of hyperandrogenism (5). A significant number of women with PCOS suffer from metabolic abnormalities (6); for example, IR is prevalent in up to 80% of women with PCOS (7). In addition, women with PCOS are more likely to experience infertility, mental problems, high blood sugar levels, dyslipidemia, hypertension, hyperinsulinemia, cerebrovascular and heart disease, liver disease, and a lower quality of life (8). Consequently, PCOS is linked to high medical, financial, and resource expenditures (9). To address the significant health challenges associated with PCOS, prioritizing research on the identification and validation of reliable therapeutic biomarkers is essential.

Met is one of the most crucial first-line therapeutic agents for diabetes (10). Clinical research has shown that Met helps PCOS patients with hyperandrogenism, menstrual irregularities, and anovulation (11, 12). According to PCOS diagnostic and care guidelines, Met has also been indicated for obese women with PCOS who have metabolic abnormalities (13). Emerging evidence from recent studies suggests that Met exhibits diverse therapeutic properties in various immune-mediated diseases, including PCOS (14, 15). These findings underscore Met’s immunomodulatory potential, yet also reveal gaps in our understanding of its precise mechanisms and clinical translatability, particularly in diverse PCOS phenotypes.

Small biologically active molecules called cytokines control and influence cell migration during inflammation, immunological surveillance, and the onset and progression of illness (14, 16–21). Throughout each immunological response, the synthesis of both pro- and anti-inflammatory cytokines is essential (22). Interleukins (ILs), pro-inflammatory cytokines, are released by monocytes, macrophages, and T cells and exacerbate inflammation. However, T cells also generate pro-inflammatory cytokines that lead to pain and tissue damage, whereas anti-inflammatory cytokines alleviate inflammation (19).

Met has been demonstrated to have direct anti-inflammatory effects in PCOS by blocking the activation of nuclear factor κB (NF-κB), triggering the synthesis of inflammatory cytokines like IL-6 (23). In addition, Met reduced the prevalence of infertility in women with PCOS while lowering inflammatory mediators such as tumor necrosis factor-alpha (TNF-α) and IL-6 (24). Furthermore, a recent study investigated how Met influences immune system development in individuals with PCOS by analyzing longitudinal changes in serum cytokine levels (pro and anti-inflammatory) (25). The findings revealed that Met use in individuals with PCOS induces a broad systemic immune activation characterized by increased levels of various cytokines. Thus, Met’s potential as a modulator of immune function in the context of PCOS. Thus, it is reasonable to hypothesize that Met plays a crucial role in regulating and suppressing the inflammatory microenvironment characteristic of PCOS by modulating both pro- and anti-inflammatory cytokines.

This review critically evaluates the molecular mechanisms by which Met modulates pro- and anti-inflammatory cytokines in PCOS, guided by three central aims: to elucidate the key molecular pathways through which Met regulates pro-inflammatory cytokines such as IL-6, TNF-α, and IL-1β, as well as anti-inflammatory cytokines like IL-10; to assess the consistency and translatability of these immunomodulatory effects across diverse PCOS phenotypes and experimental models; and to explore the therapeutic potential of combining Met with other anti-inflammatory agents in the management of PCOS.

## 2 Methods

To synthesize current evidence on the immunomodulatory role of Met in PCOS, a narrative synthesis was performed based on a systematic literature search. Electronic databases (PubMed, SciFinder, Google Scholar, Web of Science) were searched for articles published between January 2004 and November 2025. Key search terms included: “metformin,” “polycystic ovary syndrome,” “PCOS,” “cytokine,” “interleukin,” “TNF,” “TGF,” “chemokine,” and “inflammation.” This narrative review is based on a systematic literature search, but it does not constitute a formal systematic review or meta-analysis.

### 2.1 Literature screening and selection

Two authors independently screened titles and abstracts for relevance, followed by a full-text review of eligible articles. Studies were included if they met the following criteria (1): Focused on PCOS (clinical or preclinical models); (2) Investigated Met treatment or exposure; (3) Reported quantitative or qualitative data on cytokines or chemokines; and (4) Were published in English as original research or relevant reviews. Articles were excluded if they lacked cytokine data, did not focus on PCOS, or were duplicates.

### 2.2 Data synthesis

Eligible studies were categorized by cytokine type, study model (clinical/preclinical), and affected molecular pathways. Findings were narratively synthesized to evaluate mechanistic insights, consistency across studies, and clinical implications. A summary of the search and selection process is provided in **Table 1**, while the impact of Met on key cytokines, associated pathways, and clinical relevance is summarized in **Supplementary Table 1**.

**Table 1:** Outline of the search methodology.

Components	Outline
Search duration (including month and year)	September 2025 – November 2025
Databases and alternative information sources searched	PubMed, SciFinder, Google Scholar, and Web of Science
Timeframe	2004 – 2025
Relevant keywords	Metformin, PCOS, cytokine, interleukin, TNF-α, TGF-β, chemokine, inflammation
Screening process	• Two independent authors • Title/abstract screening • Full-text screening • Discrepancies resolved by consensus or a third author
Inclusion criteria	• PCOS focus (clinical and preclinical) • Metformin treatment • Cytokine/chemokine data reported • English language • Full-text research articles, reviews
Exclusion criteria	• Non-PCOS models • No cytokine outcomes reported • Non-English • Insufficient data • Duplicate or non-relevant literature that did not include information specifically for PCOS, metformin, and cytokines
Final included article	113
Data synthesis approach	Narrative synthesis, organized by cytokine and pathway

## 3 Met’s modulation of cytokines (pro and anti-inflammatory) in PCOS

Guided by the three central aims outlined in the introduction, this section systematically examines how Met modulates inflammatory signaling in PCOS. Specifically, we first delineate the molecular mechanisms through which Met regulates key pro-inflammatory and anti-inflammatory cytokines. We then critically assess the consistency of these effects across different experimental models and PCOS phenotypes. Finally, where applicable, we highlight evidence supporting combinatorial therapeutic strategies involving Met and other anti-inflammatory or metabolic agents. This framework provides a coherent basis for understanding Met’s immunomodulatory actions in PCOS.

### 3.1 IL-1 family and Met

Recent studies indicate that IL-1, a pro-inflammatory cytokine, is overexpressed in women with PCOS (26). Its ovarian expression is linked to ovarian function and pathophysiology in both PCOS women and models. Guo et al. (27) discovered that the pioglitazone Met complex formulation may alleviate PCOS by reducing inflammation, inhibiting NLRP3 inflammasome activation, and decreasing IL-1β release. Nevertheless, further investigations are still needed to elucidate the specific role of NLRP3 inflammasomes in PCOS, and such studies may lead to new treatments and management strategies for the condition.

Beyond IL-1β, other pro-inflammatory cytokines, including TNF-α and IL-6, are also dysregulated in PCOS, further implicating chronic inflammation in the condition. Furthermore, a recent study assessed Met’s impact on cytokines in rats with PCOS. The results showed that Met treatment reduced the levels of pro-inflammatory cytokines, including IL-1β, in the ovarian tissues of dehydroepiandrosterone (DHEA)-induced PCOS rats (28). This reduction in inflammatory markers was comparable to the effects observed with other treatments, such as recombinant growth differentiation factor-9 (rGDF-9) and Cetrorelix, indicating that Met has a beneficial role in mitigating ovarian inflammation in PCOS. Further studies are warranted to explore the long-term effects of Met on cytokine profiles and its potential to improve reproductive outcomes in patients with PCOS.

In addition to ovarian inflammation, systemic inflammation—particularly in PCOS with PCOS-IR—plays a critical role in metabolic dysfunction, where IL-1 and IL-1β are key contributors. A recent study measured IL-1 and IL-1β levels in PCOS-IR rat models treated with Met. The results indicated that Met reduced the levels of IL-1 and IL-1β, which are typically elevated in the inflammatory environment of PCOS-IR (29). Indeed, Met therapy led to a substantial decrease in the production of these cytokines, demonstrating its role in moderating the inflammatory response. This reduction in IL-1 and IL-1β is important because both cytokines are involved in IR and metabolic dysregulation, and their suppression by Met helps improve insulin sensitivity and reduce inflammation. Furthermore, Met’s influence on IL-1 and IL-1β is linked to its broader effect on inflammatory signaling pathways, particularly through the NF-kB/LCN-2 signaling pathway, which is implicated in the inflammatory processes that exacerbate PCOS-IR (29). While animal models provide consistent mechanistic evidence for Met’s suppression of IL-1β, direct human clinical data—particularly on its effect on the upstream NLRP3 inflammasome pathway—remain limited. The stronger findings in preclinical studies highlight a key translational gap and underscore the need for trials that directly measure NLRP3 activity and IL-1β in patients.

Another key cytokine in PCOS-related inflammation is IL-18, a member of the IL-1 family, which has been linked to both metabolic and reproductive dysfunction in PCOS. Recent studies have revealed that women with PCOS exhibit significantly higher levels of IL-18 compared to healthy controls prior to undergoing Met therapy (30). Met treatment resulted in a substantial reduction in IL-18 levels, implying that Met successfully mitigates inflammatory activity triggered by IL-18 in PCOS women. According to a study by ELMekkawi et al. (31), taking Met for three months lowered the body mass index (BMI) and the levels of IL-18 in the blood of PCOS patients. In addition, evidence suggests that chronic low-grade inflammation plays a significant role in the pathophysiology of PCOS. Heutling et al. (32) reported elevated levels of proinflammatory cytokines, including IL-18, in women with PCOS, which were positively correlated with IR. Their study further demonstrated that Met treatment improved metabolic and endocrine parameters, restored menstrual regularity, and sometimes led to spontaneous pregnancies. Additionally, the authors observed that a six-month course of Met partially reduced elevated inflammatory and endothelial markers, highlighting its potential anti-inflammatory benefits in PCOS management. The evidence for IL-18 reduction comes primarily from human observational studies, which consistently show a correlation between Met use and decreased IL-18 levels alongside clinical improvements. However, these designs cannot definitively establish causation.

The consistency of Met’s anti-inflammatory effects has been further validated in controlled clinical trials. Al-Qadhi et al. (33) conducted a double-blind, placebo-controlled trial to examine the impact of Met on IL-18 levels in Iraqi women with PCOS. The data showed a substantial decrease in serum IL-18 levels following Met therapy. Notably, after three months of Met administration, IL-18 levels decreased from baseline in the treatment group. In contrast, the placebo group showed no significant change in IL-18 levels. These findings suggest that Met not only improves insulin sensitivity but also exerts anti-inflammatory effects in PCOS patients, as evidenced by the marked reduction in IL-18, a proinflammatory cytokine associated with chronic inflammation in PCOS. This placebo-controlled trial provides higher-quality evidence for a direct anti-IL-18 effect of Met in PCOS patients, strengthening the case made by earlier observational reports.

The above-mentioned studies indicated that the quality and consistency of evidence for Met’s anti-inflammatory effects on the IL-1 family vary significantly by cytokine. The effect on IL-18 is supported by a progression of evidence from observational studies to a confirming randomized controlled trial (RCT). In contrast, the evidence for IL-1β reduction is more robust in animal models than in human studies, underscoring a disconnect between preclinical mechanisms and clinical validation. These inconsistencies likely arise from variations in PCOS phenotypes, treatment duration, and, most fundamentally, the model system (human clinical biomarkers versus animal tissue measurements). Future research requires standardized inflammatory outcome measures in longer-term clinical trials to clarify these pathways.

### 3.2 IL-2 and Met

IL-2 has pleiotropic effects; however, it has been shown to have anti-inflammatory properties in several kinds of illnesses (34, 35). Research suggests that the release of this cytokine may help regulate subclinical and systemic inflammatory processes, which are often observed in women with PCOS (36).

While IL-2 may have anti-inflammatory potential, its regulation in PCOS appears context-dependent, influenced by hormonal and metabolic factors. Luchetti et al. (37) demonstrated that Met increases IL-6, a Th2 cytokine, while reversing DHEA-induced IL-6 suppression, suggesting a protective effect against hyperandrogenism-related inflammation in PCOS. However, Met did not reduce DHEA-induced IL-2 (Th1) elevation, indicating selective cytokine modulation. This may be linked to its effects on the progesterone-induced blocking factor (37) or its anti-inflammatory effects via AMP-activated protein kinase (AMPK) activation (38). By promoting a Th2-dominant environment, Met supports pregnancy and reduces inflammation, but its inability to suppress IL-2 suggests limitations in resolving Th1-driven immune dysregulation. This finding, from a DHEA-induced rat model, illustrates that in a hyperandrogenism-dominant context, Met’s immunomodulation may favor a Th2 shift without suppressing Th1 markers like IL-2.

Interestingly, other studies report contrasting findings, with Met reducing IL-2 in specific PCOS models, highlighting variability in immune responses. Our recent study demonstrated that Met reduces elevated IL-2 levels in a C57BL/6J mouse model of PCOS, suggesting its role in modulating inflammation. However, despite this effect on IL-2, Met did not significantly alter the expression of CD2 or CD94 receptors on natural killer (NK) cells in peripheral blood or spleen. While Met reduced the absolute number of splenic NK cells, it did not restore their receptor profile (39). These findings suggest that Met affects immune regulation through cytokine modulation rather than directly influencing NK cell receptor expression in PCOS. In addition, Met has been shown to exert anti-inflammatory effects in PCOS, including the suppression of IL-2 levels. In a study using high-fat-fed New Zealand Obese mice as a model for PCOS, Met treatment significantly downregulated IL-2 expression, contributing to the modulation of the inflammatory milieu associated with the condition (40). In contrast to the DHEA model, these studies in metabolic dysfunction-focused mouse models show a consistent IL-2-lowering effect of Met, highlighting how the model’s primary etiology (hyperandrogenism versus metabolic syndrome) critically shapes the observed immune response.

The conflicting data on IL-2 modulation—with reports of both increased and decreased levels—highlight the context-dependent nature of Met’s immunomodulatory effects. As illustrated by the animal studies, these discrepancies likely originate from fundamental differences in PCOS pathophysiology. They can be hypothesized to reflect distinct PCOS endophenotypes: in a hyperandrogenism-dominant context (e.g., DHEA model), Met may drive a protective Th2 shift without suppressing androgen-elevated Th1 markers like IL-2, whereas in a metabolic syndrome-dominant context (e.g., diet-induced obese models), its primary action on insulin resistance may broadly resolve inflammation, leading to IL-2 reduction. It is essential to note that all current evidence is derived from animal models of varying designs. The absence of human clinical data measuring IL-2 in response to Met represents a significant gap. Future studies should therefore stratify patients by endophenotype, alongside inflammatory and metabolic biomarkers, to clarify these divergent immune responses and optimize targeted therapy.

### 3.3 IL-6, IL-8/CXCL8 and Met

The evidence for Met’s suppression of IL-6 and IL-8 is extensive but derives overwhelmingly from preclinical models, with emerging yet less consistent data from human studies. In rodent models of PCOS, Met consistently reduces serum and tissue levels of IL-6 and downregulates key pro-inflammatory signaling pathways, including the toll-like receptor 4 (TLR4)-Myd88-NF-κB axis and the NLRP3 inflammasome. For instance, Met combined with Diane-35 lowered IL-6 and TNF-α while inhibiting TLR4-Myd88-NF-κB in ICR mice (41). Similarly, treatment with Met alone significantly decreased serum IL-6 in a DHEA-induced rat model (42), and in letrozole-induced rats, Met reduced IL-6 and suppressed NF-κB and NLRP3/caspase-1 expression (43). These findings underscore Met’s role in mitigating chronic low-grade inflammation, a hallmark of PCOS, beyond its established metabolic benefits.

**Figure 1:**
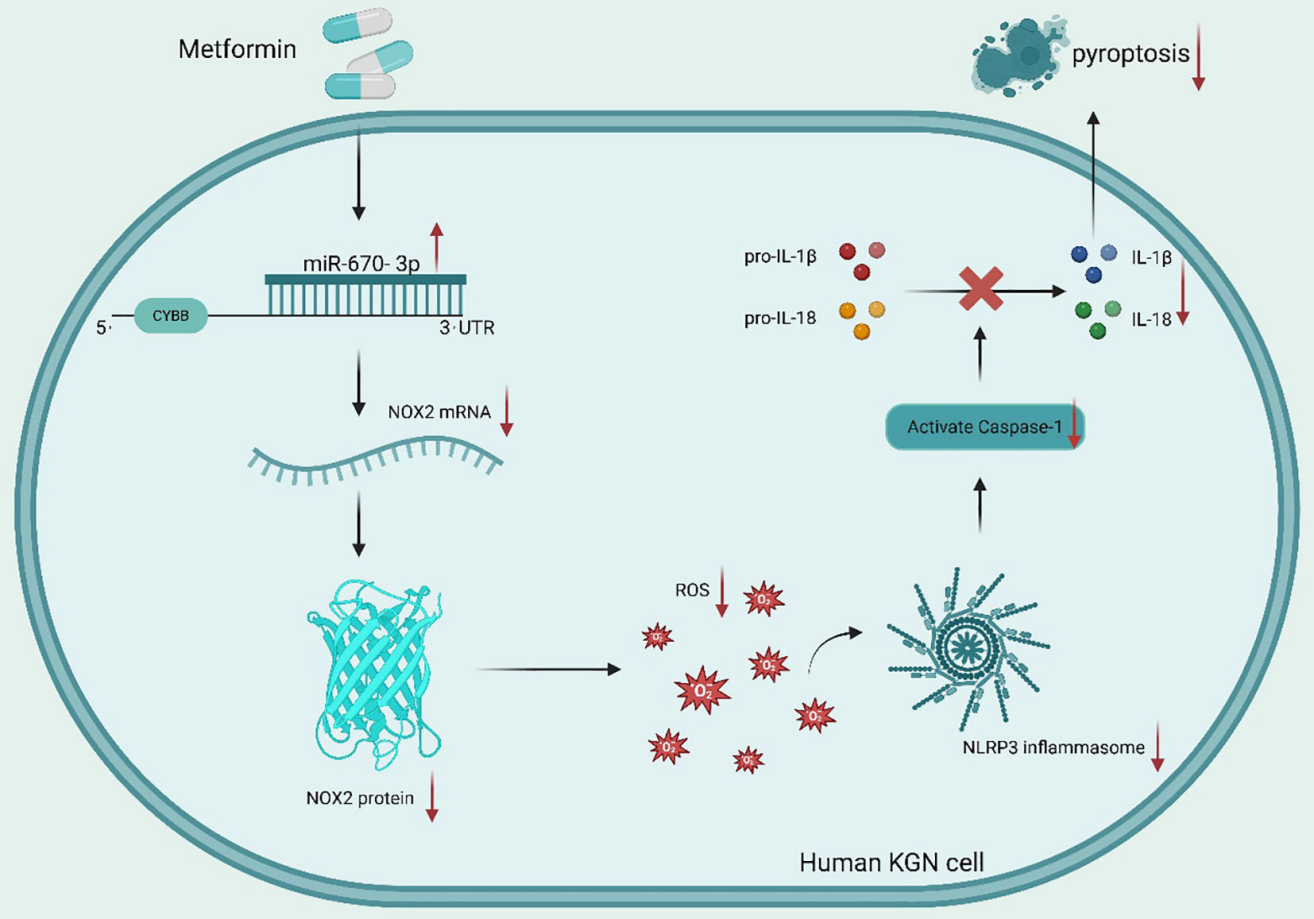
Met modulates pyroptosis in KGN Cells through the miR-670-3p/NOX2/NLRP3 pathway: insights into PCOS. Met raises microRNA-670-3p (miR-670-3p) levels, which then target the 3’ untranslated region (3’UTR) of the Cytochrome b-245 Beta Chain (CYBB) gene and suppress the production of NADPH Oxidase 2 (NOX2). This ameliorates KGN cell pyroptosis, suppresses the stimulation of the NLR Family Pyrin Domain Containing 3 (NLRP3) inflammasome, and suppresses the production of reactive oxygen species (ROS). MiR-670-3p and NOX2 may be new targets of therapy for PCOS, highlighting Met’s significance in gynecological illnesses. Red arrows (↑) indicate upregulation; blue arrows (↓) denote downregulation. Collectively, this mechanism demonstrates how Met ameliorates granulosa cell pyroptosis in PCOS by upregulating miR-670-3p, which in turn inhibits NOX2 and suppresses ROS generation and NLRP3 inflammasome activation.

Beyond cytokine modulation, Met also targets oxidative stress, a key driver of PCOS-related inflammation. Met increases the levels of miR-670-3p and subsequently reduces the transcription of the NOX2 gene. This, in turn, lowers the formation of reactive oxygen species (ROS) and activates the NLRP3 inflammasome, ultimately reducing inflammation (IL-6) and pyroptosis in human KGN cells (**Figure 1**) (44). Thus, miR-670-3p and NOX2 might be new therapeutic targets for PCOS advancement, providing additional insight into the molecular significance of Met in female reproductive disorders. This *in vitro* human cell study provides a detailed molecular mechanism linking Met to reduced IL-6 via oxidative stress pathways, bridging preclinical findings with human cell biology.

Currently, Met is increasingly being studied in combination with other compounds, particularly those targeting gut microbiota, such as probiotic inulin (a fructose polysaccharide), to enhance its therapeutic effects and improve metabolic health. Thus, in the gut microbiome of DHEA and a high-fat diet-induced PCOS mouse model, inulin and Met treatments have reduced the concentrations of pathogenic bacteria *Helicobacter* and *Parasutterella* and inflammation indicators, including IL-6 (45). Similarly, a recent study found that ovarian tissue treated with marjoram and Met had considerably lower IL-6 (46). Few clinical trials have been conducted to assess the high levels of IL-6 and IL-8 in women with PCOS. Based on an investigation by Ali et al. (47), women with PCOS had higher levels of IL-6 and IL-8, which subsequently reduced following treatment with pioglitazone and Met. In addition, a study by Liao et al. explored the effectiveness of combining glucagon-like peptide-1 receptor agonists with Met to minimize IL-6 and other molecules associated with the inflammatory immune response, as assessed through plasma proteomics (48). In a recent study by Shah et al. (49), Met and clomiphene citrate significantly reduced pro-inflammatory cytokines (e.g., IL-6). It downregulated the NF-κB pathway in a rat model of letrozole-induced PCOS. While promising, the human clinical data for IL-6/IL-8 reduction are limited to a small number of studies, some of which use combination therapies, making it difficult to isolate Met’s specific effect. The consistent findings in animal models contrast with the paucity of robust, monotherapy clinical trials in humans.

As seen with other anti-inflammatory compounds, synergistic therapies may enhance Met’s efficacy. Recent studies suggest combining Met with astaxanthin (ASX), a potent antioxidant, enhances its anti-inflammatory effects in a PCOS model (50). The combination therapy led to a more significant reduction in IL-6 and NF-κB levels than Met alone. The maximum dose of ASX (40 mg/kg) plus Met reduced malondialdehyde and raised superoxide dismutase (SOD) levels, which alleviated oxidative stress indicators and almost eliminated the expression of inflammatory cytokines (50). These findings imply that Met alone may not fully address inflammation and oxidative stress in PCOS, and ASX could enhance therapeutic outcomes. This logic has prompted research into other synergistic combinations aimed at achieving a more comprehensive therapeutic effect. In the study by Cui et al. (51), the combination of *Nasturtium officinale* (watercress) extract and Met significantly reduced pro-inflammatory cytokine IL-6 in estradiol-induced PCOS rats, showing more substantial anti-inflammatory effects than Met alone. The co-treatment also upregulated pro-apoptotic genes (Bax, p53, caspase-3), downregulated anti-apoptotic Bcl-2, and increased antioxidant enzyme activity (SOD, CAT), suggesting synergistic effects in reducing inflammation, oxidative stress, and promoting apoptosis in cystic follicles. This highlights the potential of *Nasturtium officinale* and Met in improving PCOS-related ovarian dysfunction. Together, these preclinical studies underscore the promise of combination therapies; however, further clinical studies are needed to confirm these benefits in humans.

In a study investigating the effects of gliclazide and Met on obesity-induced infertility, the combination of these drugs demonstrated significant efficacy in reducing inflammatory markers (e.g., IL-6 and NF-kB) and modulating oxidative stress biomarkers (52). The recurrent theme across preclinical research is that Met’s anti-inflammatory effect on IL-6 is often amplified in combination with other agents, suggesting its standalone action may be moderate and context-dependent.

Met’s impact on inflammation is also evident in reproductive tissues, influencing endometrial and ovarian function. In the study by Xiong et al. (53), Met was found to modulate cytokine expression in endometrial stromal cells during EP (estradiol and progesterone)-induced decidualization. EP stimulation increased IL-8, GM-CSF, and RANTES secretion while reducing IL-6 levels. Met further enhanced IL-8 and GM-CSF secretion, reversed the suppression of IL-6, and reduced RANTES levels. These effects were linked to Met’s activation of the p38-MAPK signaling pathway and Met’s downregulation of progesterone receptor (PGR) expression. The authors discussed these findings in the context of PCOS, emphasizing that Met’s regulation of cytokine secretion and signaling pathways may improve endometrial dysfunction in PCOS by modulating inflammatory and immune responses during decidualization.

Met has been shown to reduce the production of IL-8 and GROα (growth-regulated oncogene alpha) in TNF-α-stimulated human granulosa cells (GCs) by activating the AMPK pathway (54). These *in vitro* studies on human endometrial and granulosa cells provide crucial tissue-specific mechanistic insights, demonstrating Met’s direct action on key reproductive cells involved in PCOS pathophysiology.

Met consistently reduces IL-6 in preclinical models, but human data are less uniform. This discrepancy highlights a significant translational challenge. The strong, consistent evidence from animal and *in vitro* studies has not yet been matched by an equally robust body of high-quality clinical evidence. Combination therapies (e.g., with antioxidants or probiotics) often show enhanced effects in models, suggesting that Met alone may be insufficient for robust anti-inflammatory outcomes in certain subgroups. The clinical relevance of these combinations warrants validation in large, well-designed trials. Future research must prioritize controlled human studies that measure these cytokines directly to confirm if the promising preclinical findings translate to the clinic.

### 3.4 IL-17 and Met

IL-17A is the predominant cytokine released by Th17 cells. Chronic inflammatory illnesses have been associated with increased plasma concentrations of IL-17A (55). IL-17 has significance for regulating the inflammatory response because it recruits neutrophils to inflammatory regions and causes the release of pro-inflammatory mediators (56). Evidence for Met’s modulation of IL-17 in PCOS comes from a limited number of human studies, providing promising but preliminary clinical data.

Emerging research suggests Met may counteract this Th17-driven inflammation by modulating the immune balance in PCOS patients. A previous study discusses the effects of Met combined with clomiphene on immune regulation in PCOS patients. It shows that Met significantly increases serum levels of IL-10 and transforming growth factor beta (TGF-β) while decreasing IL-17 levels after treatment. IL-10 and TGF-β1 are anti-inflammatory cytokines associated with Treg cells, which help suppress inflammation and improve immune tolerance (57). In contrast, IL-17, a pro-inflammatory cytokine linked to Th17 cells, is reduced, suggesting decreased inflammation. The study also reports an increase in Foxp3 mRNA expression (a Treg marker) and a decrease in RORγt mRNA expression (a Th17 marker), indicating a shift toward an anti-inflammatory, Treg-dominant immune profile (57). This human study provides direct clinical evidence that Met can reduce IL-17 and restore the Th17/Treg balance in PCOS patients. However, as it involved combination therapy with clomiphene, the specific contribution of Met requires further isolation in controlled trials.

Met shows promise in rebalancing the Th17/Treg immune response in PCOS by reducing pro-inflammatory IL-17 and boosting anti-inflammatory IL-10 and TGF-β. This shift may help alleviate chronic inflammation and improve metabolic dysfunction. While the initial human finding is promising, it stands in contrast to the extensive preclinical evidence available for other cytokines like IL-6. There is a notable lack of supporting animal models or *in vitro* studies specifically investigating Met’s effect on IL-17 in PCOS, which limits mechanistic understanding. However, current research has significant limitations, such as the long-term clinical impact of these immunological changes, which requires further investigation. Future studies should prioritize replicating this IL-17 finding in larger placebo-controlled human trials and developing preclinical models to elucidate the underlying molecular pathways.

### 3.5 Interferons and Met

IFN has a dual function in controlling inflammation, and depending on the circumstances, it is essential to modify either pro- or anti-inflammatory activity (58). The evidence linking Met to IFN regulation in PCOS is primarily mechanistic and derived from focused *in vitro* and tissue-based research, rather than broad clinical cytokine measurements.

**Figure 2:**
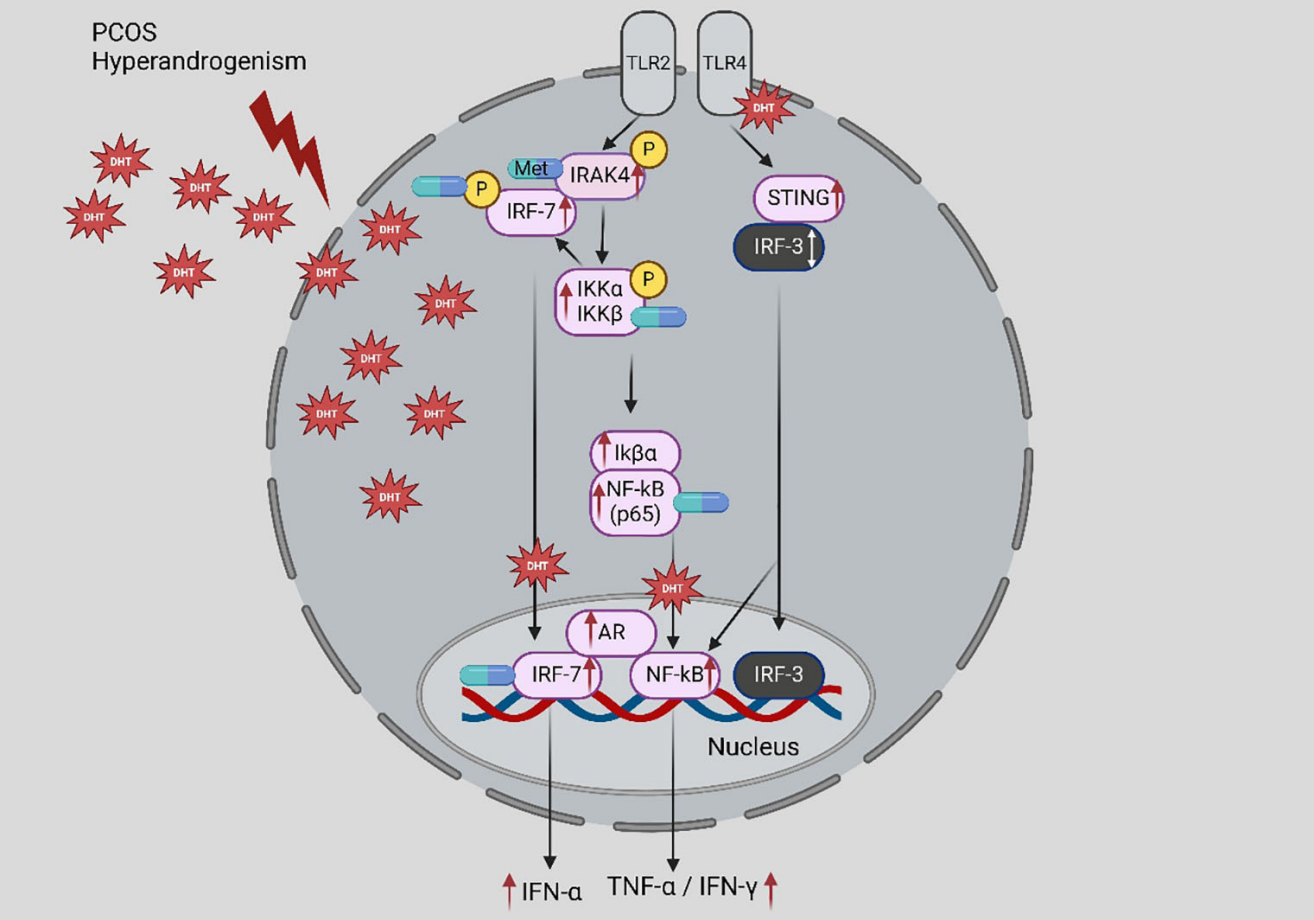
Potential avenues that, following androgen stimulation, cause the PCOS endometrium to release various cytokines and activate the nuclear factor κB (NFκB) and interferon regulatory factor 7 (IRF-7) signaling pathways. The androgen–androgen receptor (AR) axis in hyperandrogenism-afflicted PCOS patients triggers the IRF-7 and NFκB signaling pathways, which raise proinflammatory and type I interferon cytokines and endometrial inflammation, ultimately leading to endometrial cell failure. Meanwhile, androgens contribute to the activation of IRF-7 and NFκB signaling and positively influence the protein production of toll-like receptor 4 (TLR4) but not TLR2. Met may reduce endometrial inflammation in PCOS patients with endometrial hyperplasia through these mechanisms. This androgen-driven inflammatory cascade highlights how the AR axis activates NF-κB and IRF-7 pathways, promoting a pro-inflammatory endometrial environment that Met may help to mitigate.

In women with PCOS, the expression of cytokines (IFNα/γ and TNF-α) and endometrial inflammation is elevated. These conditions are associated with the androgen-induced TLR4/interferon regulatory factor 7 (IRF-7)/NF-κB signaling. In the endometrium of PCOS women, Met therapy has been demonstrated to inhibit the TLR4/IRF-7/NFκB signaling pathway (**Figure 2**) (59). This work shows that endometrial inflammation is generated and progressed by androgen-regulated innate immune response signaling and that treating endometrial innate immunity pathways is a potential PCOS therapy approach. Met seems to function as an inhibitor of androgen-triggered activation of the IRF-7/NF-κB signaling pathways, resulting in changes in the production of proinflammatory cytokines in individuals with PCOS. These findings hold significant clinical implications in this context.

This research provides a valuable tissue-specific mechanism, suggesting Met can suppress a key signaling pathway (TLR4/IRF-7/NF-κB) that drives IFN production in the PCOS endometrium. However, it is crucial to distinguish this mechanistic pathway evidence from direct clinical evidence of altered circulating IFN levels. The study’s strength lies in identifying a plausible molecular target, but it does not establish that Met consistently lowers IFN-α or IFN-γ levels in the serum of PCOS patients. This represents an important gap between tissue pathway analysis and systemic cytokine profiling.

### 3.6 TGF family and Met

The multifunctional cytokine TGF is expressed in almost every cell and tissue type. Important cellular reactions for immunological balance, wound healing, tissue homeostasis, and embryonic development are triggered by its signal transduction (60).

**Figure 3:**
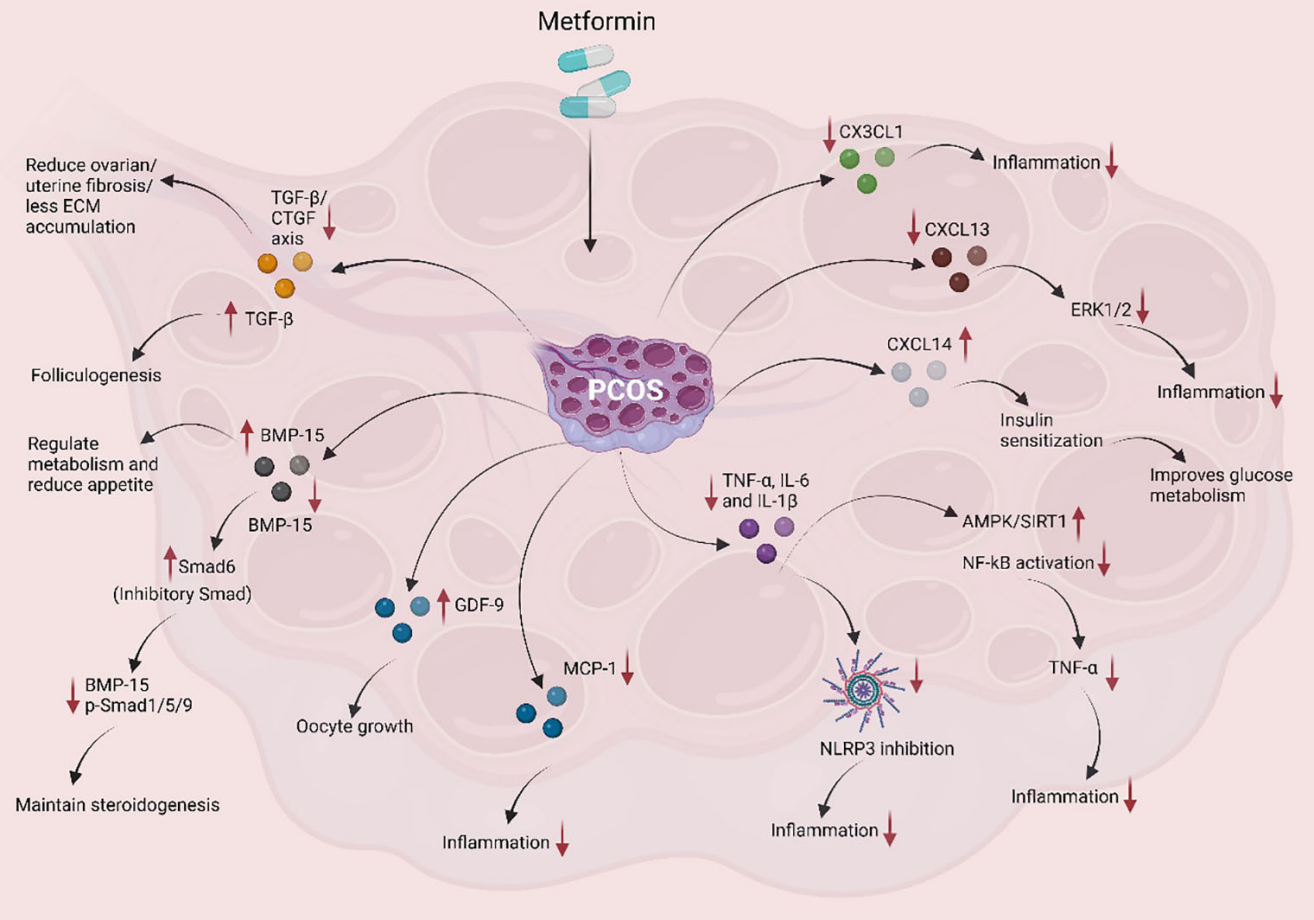
The schematic illustrates the immunomodulatory and metabolic effects of Met in the context of PCOS. Met targets multiple inflammatory and metabolic pathways to alleviate complications associated with PCOS. It significantly reduces the levels of pro-inflammatory cytokines such as TNF-α, IL-6, and IL-1β, thereby suppressing systemic inflammation. Additionally, Met inhibits the activation of the NLRP3 inflammasome, further contributing to the attenuation of inflammatory responses. In the chemokine signaling axis, Met downregulates CX3CL1 and CXCL13, MCP-1, which are associated with inflammation, while upregulating CXCL14, which enhances insulin sensitivity and improves glucose metabolism. This insulin-sensitizing effect is mediated through the activation of the AMPK/SIRT1 pathway and inhibition of NF-κB signaling, leading to decreased TNF-α production and reduced ERK1/2-mediated inflammatory signaling. Moreover, Met modulates members of the TGF-β superfamily. It downregulates the TGF-β/CTGF axis, thereby reducing ovarian and uterine fibrosis and limiting extracellular matrix (ECM) accumulation. Despite its profibrotic role, TGF-β is also essential for folliculogenesis. Met’s influence on BMP-15 (Bone Morphogenetic Protein-15) and GDF-9 (Growth Differentiation Factor-9), both crucial for oocyte maturation, further supports reproductive health in PCOS. By increasing GDF-9 expression and regulating BMP-15 activity through Smad6 (an inhibitory Smad), Met facilitates the maintenance of steroidogenesis and supports oocyte development. As depicted, Met exerts multi-target effects in PCOS, reducing pro-inflammatory cytokines and NLRP3 activation, improving chemokine profiles and insulin sensitivity via the AMPK/SIRT1 pathway, and finely regulating TGF-β superfamily members to limit fibrosis while supporting folliculogenesis.

Research into Met’s interaction with the TGF-β family in PCOS is predominantly preclinical, revealing a complex, context-dependent dual role that varies by tissue process (folliculogenesis versus fibrosis). Met has been shown to positively influence the expression of key members of the TGF-β family in PCOS follicles, which are crucial for oocyte maturation and developmental competence. In the study by Ghasemian et al. (61) Met treatment significantly upregulated the expression of TGF-β family members—including Tgfb1, Gdf9, and bone morphogenetic protein 15 (Bmp15)—in female NMRI mice with PCOS compared to untreated controls. These genes play vital roles in folliculogenesis and oocyte quality, and their upregulation following Met treatment suggests a restoration of normal follicular function (**Figure 3**). Additionally, Met reduced the expression of *Bmpr1a*, a gene associated with negative regulation of oocyte maturation, further indicating its potential to improve oocyte quality and developmental outcomes in PCOS. These findings highlight Met’s role in modulating TGF-β signaling pathways, thereby enhancing oocyte competence and supporting better reproductive outcomes in PCOS patients. This animal study demonstrates that in the context of follicular development, Met acts to upregulate beneficial TGF-β family genes, which are essential for oocyte competence.

In contrast to Met’s up-regulatory effects on folliculogenesis, other research shows it can also suppress TGF-β expression, particularly in fibrotic contexts of PCOS Morsi et al. (62) reported that Met significantly reduced TGF-β immunoexpression in ovarian tissues of letrozole-induced PCOS rats, leading to decreased fibrosis. Treated rats showed lower TGF-β levels, especially in granulosa cells, along with improved ovarian architecture and reduced fibrous tissue. This anti-fibrotic effect is likely due to Met’s modulation of TGF-β signaling, which is key to extracellular matrix (ECM) remodeling (**Figure 3**). Thus, Met not only improves metabolic and hormonal profiles but also protects ovarian tissue by reducing fibrosis, highlighting its therapeutic potential in PCOS. The apparent contradiction between Met’s upregulation of folliculogenic factors and its downregulation of TGF-β is resolved by considering the biological context: the promotion of follicular development versus the inhibition of pathological fibrotic remodeling.

These seemingly opposite effects—upregulation of TGF-β family genes in folliculogenesis and downregulation in fibrotic states—suggest Met exerts a context-specific, dual regulatory role in TGF-β signaling. It enhances oocyte maturation by upregulating Tgfb1, Gdf9, and Bmp15 while simultaneously attenuating fibrosis by suppressing fibrotic TGF-β expression. Met’s context-dependent regulation of TGF-β—promoting folliculogenesis while inhibiting fibrosis—illustrates its pleiotropic actions. However, the molecular determinants of this duality remain unclear. This duality is a compelling finding from preclinical models but remains to be confirmed in human clinical studies. The evidence for these specific, opposing effects is currently confined to animal tissues. Clinically, this suggests that patient-specific factors, such as fibrosis status or ovarian reserve, may influence treatment response and should be considered in personalized therapeutic strategies.

**Figure 4:**
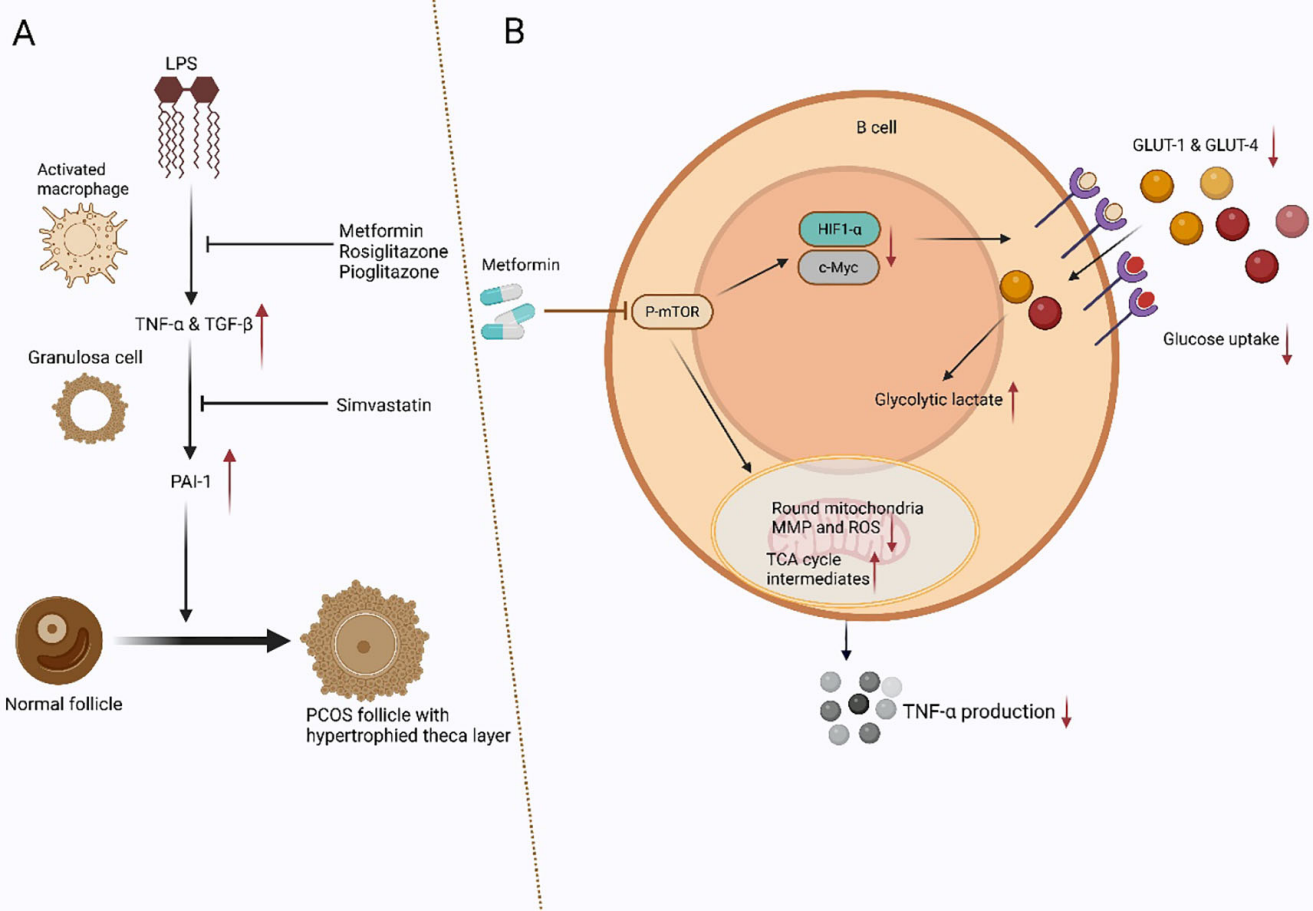
Macrophage- and B Cell-Mediated Mechanisms in PCOS: TNF-α, TGF-β, and PAI-1 Dysregulation with Therapeutic Modulation by Met, Insulin-Sensitizers, and Statins **(A)**. Hypothesis Schema: In PCOS, activated macrophages release TNF-α and TGF-β, which enhance the expression of PAI-1 in GCs. Elevated PAI-1 levels contribute to the development of polycystic ovarian morphology and thickening of the theca layer, leading to increased testosterone production. Insulin-sensitizing agents (Met, pioglitazone, and rosiglitazone) inhibit TNF-α and TGF-β production in activated macrophages, reducing PAI-1 expression in GCs. Additionally, statins directly suppress PAI-1 expression in GCs. These interactions suggest that macrophage-derived TNF-α and TGF-β increase PAI-1 expression in granulosa cells, contributing to ovarian dysfunction. This process can be targeted by insulin-sensitizing agents and statins. **(B)**. An illustration of how Met affects PCOS-related TNF-α-producing B cells. Met suppresses mTOR phosphorylation in peripheral blood B cells of women with PCOS, reduces glucose uptake by downregulating Glut1/4 and their upstream regulators HIF1α and c-Myc, alters mitochondrial morphology, and decreases ROS levels and mitochondrial membrane potential (MMP). Met enhances the accumulation of glycolytic lactate and TCA cycle intermediates while inhibiting mTOR activation in splenic B cells of DHEA-induced PCOS mice. These metabolic alterations contribute to a decrease in TNF-α production by pathogenic B cells in PCOS. Furthermore, this panel illustrates how Met rectifies metabolic dysregulation in pathogenic B cells by reducing TNF-α production through the modulation of mTOR, glucose transporters, and mitochondrial function.

Expanding this context, recent research has also explored the link between inflammation, macrophage-derived cytokines, and TGF-β-driven ovarian dysfunction in PCOS. Yamada-Nomoto et al. (63) investigated the role of plasminogen activator inhibitor-1 (PAI-1) in GCs in PCOS. They proposed that activated macrophages in PCOS produce TNF-α and TGF-β, which upregulate PAI-1 in GCs, contributing to ovarian dysfunction and hyperandrogenism (**Figure 4A**) (64–68). The study found that insulin-sensitizing drugs like Met, pioglitazone, and rosiglitazone suppress lipopolysaccharides-induced TNF-α and TGF-β expression in peritoneal fluid mononuclear cells (PFMCs), indirectly reducing PAI-1 levels in GCs (**Figure 4A**) (63). Additionally, statins like simvastatin directly inhibit PAI-1 expression in GCs. These findings suggest that combining insulin-sensitizing drugs and statins could effectively target both indirect (cytokine-mediated) and direct pathways of PAI-1 regulation, offering a potential therapeutic strategy for PCOS. This *in vitro* work on human cells (PFMCs, GCs) provides a crucial mechanistic link, showing how Met can indirectly regulate TGF-β-mediated pathways by suppressing its production in immune cells, thereby offering a unifying mechanism for its diverse effects. In addition, recent findings indicate that Met significantly downregulates TGF-β expression in endometrial tissues, which may restore endometrial receptivity by attenuating epithelial-mesenchymal transition (EMT) and fibrosis (69). Further, Met suppresses NF-κB activation, leading to a reduction in TNF-α levels, thereby mitigating the chronic inflammatory state observed in PCOS-related infertility. These findings suggest that Met exerts immunoregulatory effects, positioning it as a promising therapeutic candidate for improving reproductive outcomes in PCOS.

Further supporting Met’s anti-fibrotic capabilities, evidence shows its role in modulating the TGF-β/CTGF axis in reproductive tissues affected by PCOS. Met treatment significantly attenuated DHEA-induced fibrosis in ovarian and uterine tissues by reducing the expression of key pro-fibrotic factors, including TGF-β and connective tissue growth factor (CTGF). In DHEA-induced PCOS rats, TGF-β protein levels were markedly elevated in both ovarian and uterine tissues, suggesting its central role in fibrosis progression (70). However, Met administration led to a significant reduction in TGF-β expression, indicating its anti-fibrotic potential in PCOS (70). This effect may be mediated through the inhibition of the TGF-β/CTGF pathway, which is known to drive ECM accumulation and fibrosis. These findings underscore the role of Met, suggesting its potential as a therapeutic agent for alleviating fibrosis-related complications in PCOS. Consistent with the anti-fibrotic findings, this animal study reinforces Met’s role in downregulating the TGF-β pathway to combat tissue scarring, strengthening the preclinical case for this specific action.

The evidence for Met’s dual regulation of the TGF-β pathway is mechanistically rich but preclinically based. The upregulation of folliculogenic genes (Gdf9, Bmp15) and the downregulation of pro-fibrotic TGF-β are not necessarily inconsistent but reflect distinct biological endpoints. A key gap is the lack of human studies measuring these specific TGF-β family members in response to Met treatment. Future research should validate these context-dependent effects in patient samples to translate these promising mechanistic insights into a clinically actionable understanding.

#### 3.6.1 BMP, GDF9/15 and Met

The TGF-β superfamily includes BMP-15 and GDF-9, essential for controlling ovarian function. Especially, GDF-9 and BMP-15 are necessary for enhancing the expression of cumulus marker genes. These elements are crucial to the growth of cumulus-oocyte complexes, the growth and differentiation of cumulus cells (CCs), the synthesis of hyaluronan, and the proper functioning of GCs—all of which are critical for successful ovulation (71).

Conversely, GDF-15 is functionally distinct within the TGF-β family, acting as a stress-responsive cytokine associated with inflammation and tissue damage. GDF-15 is a cytokine that responds to stress and is linked to inflammation and tissue damage. Elevated levels have been associated with the prognosis and progression of cardiovascular disease, endothelial dysfunction, and hyperglycemia (72). Emerging evidence suggests that Met enhances progesterone synthesis in GCs by modulating BMP-15 signaling. Specifically, Met appears to impair BMP-15-induced Smad1/5/9 phosphorylation, at least in part, through the upregulation of the inhibitory Smad6 protein (73). This regulatory mechanism indicates that Met may contribute to maintaining steroidogenesis in the ovaries of women with PCOS by modulating endogenous BMP signaling pathways. These findings provide valuable insights into the broader immunometabolic effects of Met and its potential therapeutic role in restoring ovarian function in PCOS. These mechanistic insights into BMP-15 signaling are derived from *in vitro* studies on granulosa cells, providing a cellular-level explanation for Met’s potential effects on ovarian steroidogenesis.

In addition, combinatory therapies have been explored to enhance these regulatory effects further. Recent studies have investigated the impact of sitagliptin/Met (sitaformin), Met, and sitagliptin on ovarian function in women with PCOS (74). Notably, treatment with sitaformin led to a significant increase in both mRNA expression and protein levels of GDF9 and BMP15 in CCs compared to Met and sitagliptin alone. Furthermore, the expression levels of GDF9 and BMP15 mRNA positively correlated with fertilization rates and the number of grade I embryos (74). These findings suggest that sitaformin may offer superior benefits in improving oocyte quality and embryonic development in PCOS patients by enhancing the expression of key ovarian regulatory factors. Moreover, studies examining the expression of BMP-15 and GDF-9 in oocytes of PCOS patients treated with Met showed that BMP-15 protein levels remained unchanged. However, the expression of GDF-9 in oocytes was significantly upregulated following Met treatment (75). This evidence from human clinical studies—analyzing cumulus cells and oocytes from PCOS patients undergoing fertility treatment—represents a higher level of translational data. It directly shows that Met, especially in combination, can upregulate these critical fertility factors in women, linking molecular findings to potential clinical outcomes like embryo quality.

Beyond reproductive signaling, GDF-15 also plays a critical role in metabolic regulation, particularly under the influence of Met. GDF-15, a cytokine linked to metabolic and inflammatory disorders, has been implicated in PCOS, with higher levels observed in affected women and further elevation following Met treatment (76). Bioinformatics analysis reveals interactions between GDF-15 and key proteins involved in PCOS, including estrogen response, oxidative stress, interleukins (IL-18, IL-4), AGE/RAGE, leptin, TGF-β, adipogenesis, and insulin signaling (76). These findings highlight GDF-15 as a potential mediator in PCOS, warranting further research to explore its mechanistic role and therapeutic potential, particularly in the context of Met treatment and metabolic regulation. The data on GDF-15 in PCOS present an apparent paradox: it is elevated in the condition and further increased by Met, which is generally beneficial. This contrasts with the typical pattern where a harmful inflammatory cytokine is reduced by treatment. In addition, in another study, Met therapy is associated with increased plasma GDF-15 levels, likely due to its effects on mitochondrial metabolism and renal GDF-15 secretion. The study demonstrated a significant correlation between Met dosage and GDF-15 levels, suggesting a dose-dependent effect (77). Met’s inhibition of mitochondrial respiratory-chain complex 1 may trigger metabolic adaptations that enhance energy homeostasis. Additionally, the kidney has been identified as a key site of Met-induced GDF-15 secretion, which acts via the glial cell line-derived neurotrophic factor receptor alpha-like receptor in the brainstem to regulate metabolism and reduce appetite (77). These findings suggest that GDF-15 could serve as a biomarker for monitoring Met’s metabolic effects in IR and PCOS treatment. Met’s paradoxical elevation of GDF-15, a metabolic stress hormone, resolves the apparent contradiction: it is a mechanism for its anorectic and insulin-sensitizing effects, not a pro-inflammatory signal. Interestingly, GDF-15 levels may also influence appetite regulation and body weight in adolescents with PCOS. In a study by De Zegher et al. (78), adolescents with PCOS exhibited a relative deficiency of GDF15, a hormone involved in appetite regulation and weight control, despite elevated levels of its secretagogues and insulin. This deficiency suggests an impairment in the protective mechanism of appetite attenuation, potentially contributing to the excess weight gain and difficulty in weight loss commonly observed in these patients. Notably, the GDF15 deficiency persisted after treatment with oral contraceptives, a standard PCOS management approach, but was ameliorated with combined therapy of spironolactone, pioglitazone, and Met (SPIOMET), which increased GDF15 levels (78). This clinical study in adolescents introduces further complexity, suggesting a GDF-15 deficiency in a specific PCOS subgroup that can be corrected by a particular drug combination including Met. This highlights significant heterogeneity in GDF-15 dynamics across different PCOS populations and treatment regimens.

The evidence for Met’s effects on BMP-15, GDF-9, and GDF-15 spans from cellular mechanisms to human clinical data, revealing distinct narratives for each factor. For BMP-15 and GDF-9, the story is relatively consistent: Met appears to support ovarian function by modulating their expression, with stronger evidence in human cumulus/oocyte studies for GDF-9. For GDF-15, the picture is more complex and context-dependent; it may act as a beneficial, Met-induced metabolic hormone in adults while being deficient in some adolescent populations. This stark contrast underscores that the function of GDF-15 must be interpreted within its specific biological and clinical context, using it as a paradigm for the precision medicine approach needed in PCOS.

### 3.7 TNF and Met

A complex proinflammatory cytokine, TNF, is known to have a key role in the etiology and outcome of several illnesses, such as diabetes and cancer (16, 19, 20, 79). The evidence for Met’s inhibition of TNF-α in PCOS is extensive and consistent across numerous preclinical studies, though findings on its ultimate impact on ovarian follicle development are mixed, and high-quality human trial data are still limited. In a recent study, Met demonstrated significant effects on reducing inflammatory cytokines and improving hormonal balance in DHEA-induced PCOS model rats. In particular, similar quercetin and Met dramatically reduced inflammatory mediators IL-6, TNF-α, and IL-1β levels in ovarian tissue (80). Additionally, Met reduced serum levels of testosterone (T), estradiol (E2), and luteinizing hormone (LH) while increasing follicle-stimulating hormone (FSH) levels, thereby improving the LH/FSH ratio (80). These findings suggest that Met alleviates inflammation and hormonal imbalances associated with PCOS, contributing to the restoration of normal ovarian function and ovulation.

Met significantly alleviates systemic inflammation in PCOS, extending beyond its hormonal regulatory effects. As a widely used insulin-sensitizing agent, it reduces pro-inflammatory cytokines such as TNF-α and IL-1β in ovarian tissues while improving key insulin resistance markers, including serum insulin, glucose, and HOMA-IR (81). The concurrent reduction in inflammation and oxidative stress contributes to the restoration of ovarian function, underscoring Met’s dual role in modulating both metabolic and immune dysfunctions in PCOS.

However, Met’s anti-inflammatory action is tissue-specific. In the liver, it suppresses TNF-α and IL-1β by inhibiting the NLRP3 inflammasome pathway, leading to decreased activation of caspase-1 and ASC (82). In contrast, in the kidney, Met paradoxically increases caspase-1 and ASC expression, suggesting a potential exacerbation of local inflammasome activity (82). This organ-specific response highlights the complexity of Met’s immunomodulatory effects, which effectively mitigate hepatic inflammation while possibly promoting renal inflammation—a consideration of particular relevance for PCOS patients with pre-existing kidney disease.

Combination therapies may enhance Met’s anti-inflammatory benefits. A randomized trial involving 40 women with PCOS investigated the impact of a 12-week treatment with Met alone or in combination with aerobic exercise (83). The results suggest that aerobic exercise may enhance Met’s anti-inflammatory effects, as the combination treatment led to more significant reductions in TNF-α and IL-6 levels than Met alone. This study provides valuable human clinical data supporting the anti-TNF-α effect of Met, with the added insight that its efficacy can be augmented by lifestyle intervention.

Similarly, synergistic effects have been observed with other therapeutic agents. Furat Rencber and colleagues (84) examined the possible synergistic therapeutic effects of combined Met and Resveratrol (RSV) therapy in PCOS through AMPK and sirtuin 1 (SIRT1) activation. These findings indicated that the combined treatment of Met and RSV may enhance weight gain, hormone profile, and ovarian follicular cell architecture by activating SIRT1 and AMPK, which induce antioxidant and anti-inflammatory responses (decrease TNF-α). Thus, future studies should focus on clinical trials to validate the efficacy and safety of combined Resveratrol and Met therapy in PCOS patients.

Despite its metabolic benefits, Met’s impact on ovarian follicle development remains inconsistent. In recent research, Met was used as a treatment for a PCOS rat model, utilizing female Wistar rats (*Rattus norvegicus*), to evaluate its effects on IR, glucose levels, TNF-α expression, and follicle count. The findings revealed that Met significantly reduced both insulin and glucose levels compared to the PCOS control group, underscoring its efficacy in enhancing insulin sensitivity and glycemic control (85). Furthermore, Met treatment reduced TNF-α expression, highlighting its potential role in mitigating inflammation associated with PCOS. However, no significant difference was observed in follicle count between the Met-treated group and the PCOS control group, suggesting that while Met effectively improves metabolic parameters, its impact on folliculogenesis may be limited in this model (85). This study highlights a key translational disconnect: while TNF-α reduction is a consistent biomarker outcome in animal models, it does not always correlate with improved structural ovarian outcomes like follicle count, suggesting inflammation is one of several drivers of ovarian dysfunction. Although Met improves metabolic and inflammatory parameters, its inconsistent effects on folliculogenesis suggest that inflammation may not be the sole driver of ovarian dysfunction in PCOS. This underscores the need for adjunctive therapies targeting structural ovarian changes in addition to metabolic and immune pathways.

Met has been shown to exert immunomodulatory effects in PCOS by targeting TNF-α-producing B cells through metabolic reprogramming. Studies indicate that Met reduces TNF-α production in B cells derived from PCOS patients and DHEA-induced PCOS mouse models (**Figure 4B**) (86). This effect is mediated by alterations in mitochondrial function, including changes in mitochondrial morphology, decreased membrane potential, reduced ROS production, and inhibition of glucose uptake. These metabolic shifts are linked to suppressing rapamycin (mTOR) phosphorylation, a central regulator of cellular metabolism and inflammatory responses (86). Furthermore, Met treatment has been associated with improvements in PCOS phenotypes in mouse models, including the restoration of normal estrous cycles, a reduction in cystic follicles, and enhanced glucose tolerance (86). These findings suggest a novel mechanism by which Met alleviates PCOS symptoms, providing insight into its role in modulating immune and metabolic pathways. This research connects preclinical and human evidence by including *ex vivo* studies on patient-derived B cells, offering a direct cellular mechanism for Met’s anti-TNF-α effect relevant to human biology.

In addition, emerging evidence highlights the immunoregulatory role of Met in hyperandrogenized states, particularly in preclinical PCOS models. In hyperandrogenized female prepubertal BALB/c mice, DHEA treatment increased serum TNF-α levels and disrupted T lymphocyte populations, reducing CD4+ and increasing CD8+ T cells in ovarian tissue and retroperitoneal lymph nodes (87). Met co-administration mitigated these effects by normalizing TNF-α levels and restoring CD4+/CD8+ T cell balance, suggesting its potential to counteract immune dysregulation in PCOS (87). Met may exert anti-inflammatory effects by modulating cytokine profiles and T-cell distribution, warranting further research into its role as an adjunct immunotherapeutic agent for PCOS management.

Further supporting its broad anti-inflammatory properties, Met influences innate immune signaling. Studies indicate that Met reduces serum TNF-α levels, a key pro-inflammatory cytokine, and downregulates TLR-4 expression in both the liver and ovary (88). Additionally, its impact on TLR-2 expression was significant only in the ovary of animals with PCOS (88). These findings suggest that Met modulates immune signaling pathways, contributing to its broader therapeutic benefits by mitigating inflammation and immune activation in metabolic disorders.

Natural compounds combined with Met may offer additional therapeutic advantages. A recent study reported that treatment with stevia aqueous extract (SAE) and Met effectively restored the estrous cycle in letrozole-induced PCOS rats while improving hormonal balance, dyslipidemia, and hyperglycemia (89). These effects were accompanied by a significant increase in ovarian antioxidant enzyme levels, including SOD and glutathione peroxidase (GPx), as well as the upregulation of key insulin signaling genes such as glucose transporter 4 (GLUT4), SIRT1, and insulin receptor. Additionally, TNF-α expression was markedly reduced (89). The recurrent theme of enhanced TNF-α suppression with combination therapies in animal models suggests that while Met is effective, its anti-inflammatory potency may be optimized in conjunction with other agents.

Met’s ability to reduce TNF-α is one of the most reproducible anti-inflammatory findings in PCOS research, strongly supported by a wealth of preclinical data and emerging human studies. However, inconsistencies arise in two key areas: 1) its tissue-specific effects (e.g., liver versus kidney), and 2) its sometimes-disconnected impact on inflammatory biomarkers versus tangible reproductive outcomes like follicle development. The evidence indicates that while TNF-α is a valid and modifiable target, its suppression alone may be insufficient to resolve all aspects of PCOS pathology, explaining the growing research focus on synergistic combination therapies.

## 4 Other cytokines or chemokines and Met

The evidence for Met’s modulation of chemokines in PCOS is emerging but less comprehensive than for core cytokines like IL-6 or TNF-α. Findings vary significantly between chemokines and are often influenced by combination therapies and specific patient subgroups. C-X-C motif chemokine ligand 13 (CXCL13) is a distinct chemokine that promotes inflammation linked to obesity. Targeting CXCL13 could offer a potential strategy for mitigating the systemic metabolic consequences of obesity (90). Our recent study demonstrated that CXCL13 and its receptor CXCR5 are upregulated in ovarian tissues of C57BL/6J mice with PCOS, contributing to inflammation and ovarian dysfunction (14). Met treatment effectively downregulated the expression of both CXCL13 and CXCR5 in the ovaries, alongside reducing serum CXCL13 levels, indicating a systemic anti-inflammatory effect. Furthermore, the study linked CXCL13/CXCR5 signaling to the extracellular signal-regulated kinase 1/2 (ERK1/2) pathway, with Met treatment decreasing ERK1/2 phosphorylation, further supporting its role in mitigating inflammation associated with PCOS (14). These findings highlight Met’s potential to modulate chemokine-mediated inflammatory pathways in PCOS, offering new insights into its therapeutic mechanisms. The evidence for CXCL13 suppression is strong but currently limited to preclinical mouse models, providing a clear mechanistic pathway but awaiting confirmation in human studies.

Beyond CXCL13, other chemokines like CXCL14 also play a role in PCOS-related metabolic dysfunction. The chemokine CXCL14, released by active brown or beige adipose tissue, has been shown to enhance glucose metabolism in rodent models of IR (91). A study investigating adolescent girls with PCOS reported significantly reduced circulating levels of CXCL14, which were restored following SPIOMET treatment—a combination of spironolactone, pioglitazone, and Met (92). While Met alone did not directly induce CXCL14 expression in human adipocytes, its insulin-sensitizing effects likely contributed to the normalization of CXCL14 levels, correlating with improvements in IR (92). This finding suggests that insulin-sensitizing therapies may indirectly regulate CXCL14 expression, highlighting a potential link between CXCL14 and metabolic dysfunction in PCOS. This study provides a key piece of human data, showing that Met, as part of a specific combination therapy (SPIOMET), can normalize CXCL14 levels in a defined adolescent PCOS population. The lack of a monotherapy effect highlights its indirect, context-dependent role.

Current clinical trials are investigating Met’s role in CXCL14 modulation through combination therapies. In an ongoing study (protocol version from 2022 to 2026), Met is a key component of the mini-spiomet combination (with spironolactone and pioglitazone), targeting ectopic fat and metabolic dysregulation in girls with early puberty (93). While results are pending, the study design suggests that the SPIOMET combination (a higher-dose version) may normalize reduced CXCL14 levels, as seen in prior studies of girls with PCOS. However, the precise mechanism by which Met influences CXCL14 remains unclear and warrants further investigation. This study highlights Met’s potential, particularly in combination therapy, to modulate CXCL14 and address IR and ectopic fat in pediatric populations.

Another key chemokine, MCP-1, demonstrates variable responses to Met treatment in PCOS. A member of the CC chemokine family, MCP-1 (monocyte chemoattractant protein-1) is often referred to as chemokine (CC-motif) ligand 2 (CCL2). It is essential to inflammation because it attracts or increases the expression of other inflammatory cells and elements (94, 95). Furthermore, the study by Sathyapalan et al. (96) investigated the effects of atorvastatin and subsequent Met treatment on inflammatory markers in overweight/obese women with PCOS. The researchers found that 12 weeks of atorvastatin significantly reduced levels of acylation-stimulating protein (ASP), IL-6, and MCP-1, markers associated with adipose tissue dysfunction and inflammation. These reductions were maintained after an additional 12 weeks of Met treatment, suggesting that Met has a sustained suppressive effect on these inflammatory markers. The findings indicate that Met, particularly when used following atorvastatin, may help mitigate chronic low-grade inflammation in PCOS, contributing to improved insulin sensitivity and reduced hyperandrogenemia.

However, not all studies demonstrate Met’s efficacy in modulating MCP-1. In a 12-month randomized study investigating the effects of Met and oral contraceptives (OCP) on inflammatory markers in women with PCOS, Met treatment, either alone or in combination with OCP, did not significantly alter levels of MCP-1 (97). Despite improvements in body composition, including reductions in regional fat mass, no significant changes in MCP-1 levels were observed (97). This suggests that while Met may improve metabolic and body composition parameters, its impact on MCP-1 chemokine levels in PCOS patients appears to be limited. The evidence for MCP-1 is directly conflicting between human clinical trials. This inconsistency may be explained by differences in study design, such as prior statin use versus no prior statin use, and patient characteristics like BMI and concomitant medications, underscoring the highly variable and context-sensitive nature of chemokine responses.

The variable impact of Met on chemokines such as MCP-1 and CXCL14 highlights the influence of patient-specific factors—including BMI, androgen levels, and concomitant medications. Future research should aim to identify biomarkers that predict chemokine response, facilitating more targeted and effective use of Met in PCOS management.

Met’s immunomodulatory effects extend to other chemokines like fractalkine (CX3CL1). Our previous research has demonstrated the critical role of maternal immune regulators, including CX3CL1, in the pathogenesis of preeclampsia (17). Interestingly, Met has also shown promising anti-inflammatory effects in PCOS by improving IR and reducing chronic inflammation. Studies report a significant decrease in serum CX3CL1 levels following Met therapy (98), which is attributed to reduced hyperinsulinemia, hyperglycemia, and reactive oxygen species production, collectively alleviating inflammation. Additionally, improvements in hirsutism, acne, acanthosis, and BMI further highlight Met’s role in addressing both metabolic and inflammatory aspects of PCOS, including modulation of the CX3CL1 signaling pathway (98). The data on CX3CL1 reduction comes from human studies, adding to the clinical evidence base. However, like other chemokines, its modulation is likely a secondary consequence of Met’s primary metabolic effects rather than a direct target.

The landscape for chemokines is notably heterogeneous compared to cytokines like IL-6 or TNF-α. For CXCL13, evidence is strong but preclinical. For CXCL14 and CX3CL1, human data exist but point to indirect, combination-dependent, or metabolic consequence-driven effects. The most striking inconsistency is for MCP-1, where directly opposing results in human trials highlight the critical influence of prior treatments and study population. This collective evidence suggests chemokines may be less reliable or consistent biomarkers of Met’s anti-inflammatory action in PCOS than some core cytokines, and their response is highly contingent on the clinical and therapeutic context. **Table 2** provides a detailed overview of Met’s clinical and preclinical applications, highlighting its role in cytokine modulation for PCOS treatment.

**Table 2:** The therapeutic use of Met in clinical and preclinical studies focuses on cytokine modulation for PCOS. ↑ denotes up-regulation, ↓ signifies down-regulation, and ↔ represents no change.

Target cytokines	Study type/trial number	Dosage	Frequency	Treatment duration	Tissue/cell	Expression level of cytokines	References
IL-6/IL-18	Clinical/NCT00428311	850 mg	Twice daily	24-weeks	Blood	IL-6 ↓ and IL-18↔	(99)
IL-6/IL-8	Clinical/NCT03117517	Met (500 mg plus pioglitazone (15 mg) or Met alone	Once Daily	12 weeks	Blood	IL-6 and IL-8	(47)
IL‐6/TNF‐α	Clinical/NCT05233514	Aerobic exercise plus Met (1,500 mg)	Once Daily	12 weeks	Blood	IL‐6 and TNF‐α ↓	(83)
IL‐6/TNF‐α/IL-8	Clinical/NCT03151005	Acetate (2mg) /ethinylestradiol (35-μg) + Met (1500 mg)	Once Daily	12 weeks	Blood	IL‐6 ↓ while TNF‐α and IL-8 ↔	(48)
IL-6/MCP-1	Clinical/NCT00451568	2 g	Once Daily	12 months	Blood	↔	(97)
TNF-α/IL-6	Clinical/NCT02302326	500/1000/1500 mg	Once daily	12 weeks	Blood	IL‐6 and TNF‐α ↓	(24)
IL-17	Clinical/NCT00159536 and NCT01587378	2000 mg	Once daily	(gestational weeks 5 to 12) and continued until the delivery	Blood	IL-17 ↓	(25)
GDF9/BMP15	Clinical/NCT04268563	sitagliptin/Met (50/500 mg)	Twice a day	2 months	Follicular fluid and blood	GDF9 and BMP15 ↑	(74)
BMP15	Clinical/ISRCTN29234515 and ISRCTN11062950	A combination of 50 mg/day spironolactone, 7.5 mg/day pioglitazone, and 850 mg/day Met	once daily at dinner-time	1 year	Blood	BMP15 ↑	(78)
MCP-1/IL-1β/INF-γ/IL-6/IL-7, IL-10/IL12/TNF-α	Clinical/ISRCTN58369615 and ISRCTN75758249	500 mg	Once Daily	12 weeks	Blood	↔	(100)
GDF-15	Clinical	1,500 mg	single nightly dose	60 days	Blood	GDF-15 ↑	(76)
IL-10/TGF-β1/IL-17	Clinical	clomiphene (50–100 mg) and Met (500 mg)	Clomiphene for 5 days per cycle (starting day 5) + Met 3 times daily	3 menstrual cycles	Blood	IL-10 and TGF-β1↑ while IL-17 ↓	(57)
IL-6/IL-18	Clinical	850mg	Not mentioned	3 months	Blood	IL-6 and IL-18↓	(31)
TNF-α/IL-6	Clinical	1500 mg	Once Daily	3 months	Blood	IL‐6 and TNF‐α ↓	(101)
IL-6	Clinical	500 mg	three tablets daily	3 months	Blood	IL-6↓	(102)
IL-18	Clinical	850 mg	Once daily	12-week	Blood	IL-18 ↓	(33)
IL-1β/IL-6/TNF-α	Clinical	15mg pioglitazone and 500mg of Met	Once daily	12-week	Blood	IL-1β, IL-6, and TNF-α ↓	(27)
IL-18	Clinical	Not mentioned	Not mentioned	3 months	Blood	IL-18 ↓	(30)
IL-6/IL-18	Clinical	850 mg	twice daily	6 months	Blood	IL-6 and IL-18↓	(32)
IL-6	Clinical	850 mg	Not mentioned	12 months	Blood	IL-6 ↓	(103)
TGF-β1	Clinical	Met (0.5 g) and cyproterone acetate/ethinylestradiol concentration not mentioned	2–3 times daily	6-months	Blood	TGF-β1↓	(104)
TNF-α	Clinical	500 mg	Three times daily	24 weeks	Blood	TNF-α ↑	(105)
TNF-α	Clinical	1275–1500 mg	Once Daily	3 months	Blood	TNF-α ↓	(106)
IL-1/β/IL-6/TNF-α/IFN-γ	Pre-clinical	500 mg	Once Daily	4-weeks	Blood	IL-1/β, IL-6, TNF-α, and IFN-γ ↓	(29)
IL-2	Pre-clinical	50 mg	Once Daily	20 days	Blood	IL-2 ↓	(39)
TNF/IL-6/IL-1β GDF9/BMP15/TGFβR1/BMPR2	Pre-clinical	50 mg	Once Daily	28 days	Ovarian	TNF, IL-6, and IL-1β↓, while TGFβ superfamily markers (GDF9, BMP15, TGFβR1, and BMPR2)↑	(107)
TNF-α/IL-6	Pre-clinical	0.5 g Met + 0.4 mg Daine-35	Once Daily	4 weeks	Blood	TNF-α and IL-6 ↓	(41)
IL-1β/IL-6/TNF-α	Pre-clinical	2 mg	Once Daily	14 days	Blood and ovarian tissue	TNF-α, IL-6 and IL-1β ↓	(43)
IL-1β/IL-6/TNF-α	Pre-clinical	100 mg	Once Daily	20 days	Ovarian tissue	TNF-α, IL-6 and IL-1β ↓	(80)
IL-6/TNF-α	Pre-clinical	20 mg marjoram +500 mg Met	Once Daily	21 days	Ovarian tissue	IL-6 and TNF-α ↓	(46)
TNF-α/IL-6/IL-17A/IL-10	Pre-clinical	1.9 g	Once Daily	21 days	Blood and ovarian tissue	TNF-α, IL-6, and IL-17A ↓ IL-10 ↑	(45)
IL-6/TNF-α/GDF9/BMP15/	Pre-clinical	Patuletin (25mg) and clomiphene citrate (50 mg) +Met (300mg)	Once Daily	28 days	Ovarian tissue	IL-6 and TNF-α ↓ While GDF9 BMP15 ↑	(49)
IL-6	Pre-clinical	300 mg	Once Daily	34 days	Blood	IL-6 ↓	(42)
TNF-α/IL-6/IL-18	Pre-clinical	0.008 g	Once Daily	28 days	Blood	TNF-α and IL-6 ↔ while IL-18 ↓	(108)
TNF-α/IL-6	Pre-clinical	Met (20 mg) and (40 mg) Astaxanthin	Once Daily	7 days	Ovarian tissue	TNF-α/IL-6 ↓	(50)
IL-1β/IL-6	Pre-clinical	MET 300 mg), *Nasturtium officinale L.* (50 and 100 mg	Once Daily	21 days	Blood	IL-1β and IL-6 ↓	(51)
IL-6	Pre-clinical	200 mg	Once Daily	21 days	Blood	IL-6 ↓	(109)
IL-6	Pre-clinical	Gliclazide (5, 10 mg) and Met (100, 300 mg)	Once Daily	28 days	Ovarian tissue	IL-6 ↓	(52)
TNF-α/IL-6	Pre-clinical	265 mg	Once Daily	4 weeks	Blood	IL-6 ↓ while TNF-α ↔	(110)
TNF-α	Pre-clinical	Met (780 mg) and Stevia leaf extract ((500 mg)	Once Daily	50 days	Blood	TNF-α ↓	(62)
TNF-α/TGF-β	Pre-clinical	.0275 g	Once Daily	21 days	Blood and endometrial tissue	TNF-α ↓ while TGF-β ↑	(69)
TGF-β	Pre-clinical	30 mg	Once Daily	35 days	Ovarian and uterine tissues	TGF-β ↓	(70)
TNF-α/IL-1β	Pre-clinical	300 mg	Once Daily	30 days	Ovarian tissue	TNF-α and IL-1β ↓	(81)
TNF-α	Pre-clinical	Met (300 mg) +Resveratrol (20 mg)	Once Daily	28 days	Blood and ovarian tissue	TNF-α ↓	(84)
TNF-α	Pre-clinical	2mg	Once Daily	21 days	Blood	TNF-α ↓	(85)
TNF-α	Pre-clinical	200 mg	Once Daily	29 days	Blood	TNF-α ↓	(86)
TNF-α/IL-6	Pre-clinical	20mg	Once Daily	21 days	Blood	TNF-α and IL-6 ↓	(111)
TNF-α	Pre-clinical	Met (300 mg) + bee pollen (200 mg)	Once Daily	21 days	Blood	TNF-α ↓	(112)
TNF-α	Pre-clinical	50mg	Once Daily	20 days	Blood	TNF-α ↓	(87)
TNF-α	Pre-clinical	500 mg	Once Daily	28 days	Blood	TNF-α ↓	(88)

## 5 Clinical implications of Met-based combination therapies

The combination therapies of Met explored for PCOS management demonstrate promising multimodal benefits, particularly in improving IR, reducing inflammation, and ameliorating hormonal dysregulation (**Table 3**). From a clinical perspective, the evidence supports a stratified approach: for adolescent and non-obese PCOS patients with marked hyperandrogenism, the SPIOMET combination (spironolactone, pioglitazone, and Met) shows a strong profile for improving insulin sensitivity, reducing visceral fat, and normalizing androgen levels, with CXCL14 emerging as a novel biomarker of response (78, 92). In cases of overweight/obese PCOS with primary metabolic dysfunction, combining Met with a GLP-1 receptor agonist (e.g., liraglutide) or pioglitazone yields superior weight loss and metabolic improvement compared to Met alone (47, 48), with aerobic exercise adding potent anti-inflammatory benefits (83). To address infertility, combinations of Met with either clomiphene or sitagliptin improve key outcomes, including ovulation rates, oocyte quality, and embryonic markers such as GDF-9/BMP-15, with Met plus clomiphene specifically shown to improve IR and oxidative stress (57, 74). Key biomarkers for monitoring therapeutic efficacy across these regimens include HOMA-IR, inflammatory cytokines (IL-6, TNF-α, TGF-β1), adipokines (e.g., CXCL14), and growth differentiation factors (GDF-9, GDF-15). To strengthen the evidence base, priority should be given to larger, blinded, RCTs with direct comparator arms, particularly for newer combinations (e.g., those involving resveratrol or gliclazide). Such trials are essential to facilitate the translation of these findings into personalized, biomarker-guided treatment protocols in clinical practice.

**Table 3:** Met combination therapy in the clinical and preclinical studies of PCOS.

Combination therapy	Study type (clinical/preclinical)	Key outcomes	Strength of evidence	References
Met plus Pioglitazone	Clinical	The combination therapy significantly reduced serum levels of IL-6 and IL-8, decreased HOMA-IR and fasting insulin, lowered luteinizing hormone, testosterone, follicle-stimulating hormone, and prolactin levels, and reduced body weight and body mass index in women with PCOS over 12 weeks, proving more effective than Met alone in reducing IL-6 and insulin resistance.	Moderate to high, based on a randomized clinical trial with 106 participants but without blinding or a pioglitazone-only control group.	(47)
Spironolactone plus Pioglitazone plus Met (SPIOMET)	Clinical (adolescent girls)	Serum CXCL14 levels were reduced in PCOS girls compared to controls. SPIOMET treatment for 1-year normalized CXCL14 levels, improved insulin sensitivity (HOMA-IR), reduced central fat, and enhanced the adipokine profile. *In vitro*, pioglitazone induced CXCL14 expression during adipocyte differentiation, and spironolactone increased CXCL14 release from mature adipocytes.	Moderate to strong; randomized controlled trials in humans with *in vitro* mechanistic support, though limited by sample size and lack of direct causal evidence linking CXCL14 normalization to metabolic improvement.	(92)
Spironolactone plus Pioglitazone plus Met (SPIOMET)	Clinical (randomized, open-label pilot studies in non-obese adolescent girls with PCOS)	Compared to oral contraceptives, the SPIOMET intervention elicited a significantly greater and sustained increase in circulating growth differentiation factor 15, concurrent with marked reductions in insulin resistance (HOMA-IR), systemic inflammation (C-reactive protein), hepatic steatosis, visceral adiposity, and androgen levels (total testosterone, free androgen index).	Moderate, based on randomized human pilot studies with biochemical and imaging endpoints, but limited by open-label design, *post-hoc* analysis of growth differentiation factor 15, and a relatively small sample size.	(78)
Aerobic exercise plus Met	Clinical (randomized controlled trial)	The dual intervention of aerobic exercise and Met resulted in significantly greater reductions in the inflammatory markers IL-6, TNF-α, and C-reactive protein in women with PCOS.	Moderate to strong, based on a randomized controlled trial with blinded assessment, though limited by a small sample size and lack of an exercise-only control group.	(83)
Sitagliptin plus Met (Stiaformin)	clinical (randomized, placebo-controlled, blinded pilot study in PCOS patients undergoing intracytoplasmic sperm injection).	Compared to monotherapy or placebo, Stiaformin improved fertility outcomes and molecular markers, including oocyte maturation rates, high-grade embryos, and growth differentiation factor 9/bone morphogenetic protein 15 expression in cumulus cells. The therapy concurrently reduced insulin resistance, free androgen index, leptin, anti-Müllerian hormone, and oxidative stress (malondialdehyde).	Moderate to good, based on a randomized, placebo-controlled, blinded human trial with molecular, biochemical, and clinical embryology outcomes, though limited by a small final sample size and being a single-center pilot study.	(74)
Met plus clomiphene	Clinical (randomized controlled trial in 94 patients with PCOS).	The combination therapy provided significant benefits over clomiphene monotherapy, including improved insulin resistance (reduced HOMA-IR, fasting insulin, and fasting C-peptide), decreased oxidative stress (lowered total oxidant status, malondialdehyde, and advanced oxidation protein products; increased total antioxidant status, superoxide dismutase, glutathione peroxidase, vitamin C, and vitamin E), and a beneficially modulated T-cell immune response (elevated forkhead box P3, IL-10, and TGF-β1; reduced retinoic acid-related orphan receptor gamma t and IL-17).	Moderate, based on a randomized controlled human trial with biochemical and immunological measurements, but limited by sample size and single-center design.	(57)
Met plus oral contraceptives (OCP)	Clinical (randomized controlled trial)	Treatment with Met alone or combined with OCP improved body composition by reducing regional fat mass compared to OCP alone, while inflammatory markers remained unchanged across all groups; OCP alone was associated with a small but significant weight gain, but did not increase inflammatory markers.	Moderate, due to the randomized controlled design, though high dropout rates and a relatively lean study population limit generalizability.	(97)
GLP-1 Receptor Agonist (Liraglutide) plus Met	Clinical	In overweight women with PCOS, 12 weeks of liraglutide plus Met therapy significantly reduced body weight, body mass index, waist circumference, fasting blood glucose, hemoglobin A1c, insulin resistance, total cholesterol, low-density lipoprotein cholesterol, and IL-6 levels while improving insulin sensitivity and ovulation rates. This regimen was more effective than cyproterone acetate/ethinylestradiol plus Met in improving metabolic parameters and ovulation, but less effective in reducing hyperandrogenemia.	Moderate, based on a randomized controlled trial with 60 participants and an open-label design, but limited by a lack of blinding and a single-center, relatively small sample size.	(48)
Resveratrol plus Met	Preclinical (rat model)	Co-administration of Met and resveratrol improved ovarian weight, hormone profiles (testosterone, luteinizing hormone, anti-Müllerian hormone), inflammation (TNF-α), oxidative stress (malondialdehyde), follicular architecture, and reduced apoptosis. This synergistic effect, associated with activation of both SIRT1 and AMPK pathways, suggests the therapy improves the PCOS phenotype by mitigating weight gain, hormonal imbalance, and follicular disruption through enhanced antioxidant and anti-inflammatory mechanisms.	Moderate; well-designed experimental study with biochemical, histological, immunohistochemical, and ultrastructural analyses, but limited to a DHEA-induced rodent model and not yet validated in humans.	(84)
Clomiphene citrate plus Met	Preclinical (rat model)	The combination therapy significantly reduced body weight in letrozole-induced PCOS rats, decreased testosterone levels, increased estradiol and progesterone levels, upregulated the expression of growth factors growth differentiation factor 9 and bone morphogenetic protein 15, downregulated inflammatory markers TNF-α and IL-6, modulated the expression of gonadotropin and steroid receptors and cytochrome P450 enzymes, and improved ovarian histology by reducing cystic follicles and promoting corpus luteum formation.	Low to moderate, based on a preclinical animal study with a small sample size per group (n=12) and no human trial data; results are promising but require clinical validation.	(49)
Nasturtium officinale L. extract with Met	Preclinical (animal model using estradiol-induced PCOS in rats).	Synergistic alleviation of PCOS symptoms via modulation of Bax/Bcl-2/p53/caspase-3 signaling pathway, reduction in pro-inflammatory cytokines (IL-1β, IL-6), enhancement of antioxidant enzymes, normalization of hormone levels, reduction in cystic follicles, and promotion of normal follicular development and ovulation.	The evidence is moderately strong, as it is based on thorough lab and tissue tests in animals, but it is not yet confirmed in human studies.	(51)
Gliclazide plus Met	Preclinical (animal model using female Wistar rats with obesity-induced PCOS).	Treatment with Gliclazide plus Met significantly reduced body weight, improved glucose tolerance and lipid profile, normalized reproductive hormones (luteinizing hormone, follicle-stimulating hormone, progesterone, estradiol, testosterone), reduced inflammatory markers (e.g., IL-6), enhanced ovarian antioxidant defenses (glutathione, superoxide dismutase, catalase), reduced oxidative stress (malondialdehyde, nitric oxide), and restored ovarian follicular architecture.	Moderate, based on a controlled animal study with comprehensive biochemical, hormonal, inflammatory, oxidative stress, and histopathological evaluations.	(52)

## 6 Conclusions and future prospectives

Met demonstrates a well-supported capacity to reduce key pro-inflammatory cytokines such as IL-6 and TNF-α, primarily through the inhibition of the NF-κB and NLRP3 inflammasome pathways. These effects are consistently observed across preclinical models and are increasingly corroborated by clinical evidence, underscoring their central role in alleviating the chronic low-grade inflammation characteristic of PCOS. Met’s modulation of cytokine dynamics also involves key signaling pathways such as AMPK and TGF-β, which are central to improving insulin resistance and reproductive outcomes. In contrast, the modulation of other cytokines, including IL-1β, IL-2, IL-17, and various chemokines (e.g., MCP-1, CXCL14), presents a more exploratory picture; findings are often inconsistent and appear highly context-dependent, influenced by factors such as PCOS phenotype, treatment duration, and concomitant therapies. Met’s combination with other therapies, such as clomiphene citrate, astaxanthin, and probiotics, has shown enhanced anti-inflammatory and metabolic benefits, suggesting its potential as part of a multifaceted treatment approach for PCOS.

While Met demonstrates clear immunomodulatory potential, the existing evidence is limited by methodological heterogeneity, small sample sizes, and a predominance of preclinical data. Translating these findings into clinical practice requires a structured research agenda organized around three key themes:

a. Mechanistic Studies.

Focus on tissue-specific signaling determinants and molecular pathways underlying Met’s immunomodulatory effects, particularly the dual role of TGF-β in folliculogenesis versus fibrosis.

b. Clinical Validation.

Conduct phenotype-stratified trials with standardized biomarker correlates (e.g., IL-6, TNF-α, GDF-15) to validate preclinical findings and link cytokine modulation to clinical outcomes.

c. Therapeutic Optimization.

Develop personalized combination regimens (e.g., Met with antioxidants, probiotics, or insulin sensitizers) and evaluate their efficacy in well-designed randomized controlled trials. By addressing these priorities, future research can bridge translational gaps and advance toward biomarker-guided, personalized Met-based therapies for PCOS.
