# Supplementary material for: Beyond glycemic control: molecular mechanisms of metformin in modulating cytokine networks in polycystic ovary syndrome
Source: Front Endocrinol (Lausanne). 2026 Feb 9;17:1749906. doi: 10.3389/fendo.2026.1749906 (PMC12926119; doi:10.3389/fendo.2026.1749906)
Supplement: Supplementary file 1 [file Table1.docx]

Supplementary Table 1: Met’s immunomodulatory effects on key cytokines in PCOS.

| **Cytokines** | **Effect of Met** | **Major Pathways Involved** | **Strength of evidence** | **Clinical implications** | **References** |
| --- | --- | --- | --- | --- | --- |
| IL‑1β, IL‑6, IL‑18, TNF‑α | ↓ | miR‑670‑3p/NOX2/ROS/NLRP3 inflammasome pathway | Moderate (preclinical—KGN cell line and PCOS mouse models; no human clinical data). | Suggests miR‑670‑3p and NOX2 as therapeutic targets for PCOS; supports Met’s anti-inflammatory mechanism in ovarian protection beyond insulin sensitization | (1) |
| TNF-α | ↓ | mTOR‑dependent metabolic reprogramming; AMPK/PI3K/mTOR signaling; mitochondrial remodeling (↓ membrane potential, ↓ ROS); glucose uptake via Glut1/Glut4/HIF1α/c‑Myc | Moderate (human patient samples, in *vitro* B‑cell studies, DHEA-induced mouse PCOS model; no randomized clinical trial reported | Identifies TNF‑α-producing B cells as a novel immune target in PCOS; supports metformin’s immunomodulatory role via mTOR‑metabolic axis; suggests potential for B-cell‑targeted therapies in PCOS | (2) |
| TNF-α and TGF-β | ↓ | VEGF signaling and TGF-β-mediated fibrosis, | Moderate (well-conducted preclinical animal study) | Reinforces Met as a multifaceted PCOS therapy targeting inflammation, fibrosis, and angiogenesis | (3) |
| IL-17 | ↓ | Th17/Treg axis (via Foxp3/RORγt) | Limited clinical (combination therapy) | Supports using Met as an adjunct to clomiphene to comprehensively improve metabolic, oxidative, and immune dysfunction in PCOS | (4) |
| GDF‑9 / BMP‑15 | ↑ | Smad1/5/9 | Strong mechanistic preclinical evidence (in vitro cell study) and clinical (human oocytes/cumulus cells) | Improves oocyte maturation, embryo quality, and ovulation outcomes. | (5), (6), (7) |

↑, upregulation ↓, downregulation. KGN: granulosa-like tumor cell line; NF‑κB: nuclear factor kappa B; NLRP3: NLR family pyrin domain containing 3, AMPK: AMP-activated protein kinase; mTOR: rapamycin; TGF‑β: transforming growth factor beta; CTGF: connective tissue growth factor; BMP: bone morphogenetic protein; GDF: growth differentiation factor.

**References**

1. Zhou LH, Zou H, Hao JY, Huang Y, Zhang JN, Xu XH, Li J. Metformin inhibits ovarian granular cell pyroptosis through the miR-670-3p/NOX2/ROS pathway. *Aging (Albany NY)* (2023) 15:4429–4443. doi: 10.18632/aging.204745

2. Xiao N, Wang J, Wang T, Xiong X, Zhou J, Su X, Peng J, Yang C, Li X, Lin G, et al. Metformin abrogates pathological TNF-α-producing B cells through mTOR-dependent metabolic reprogramming in polycystic ovary syndrome. *Elife* (2022) 11:e74713. doi: 10.7554/eLife.74713

3. Morsi AA, Mersal EA, Farrag ARH, Abdelmoneim AM, Abdelmenem AM, Salim MS. Histomorphological Changes in a Rat Model of Polycystic Ovary Syndrome and the Contribution of Stevia Leaf Extract in Modulating the Ovarian Fibrosis, VEGF, and TGF-β Immunoexpressions: Comparison with Metformin. *Acta Histochem Cytochem* (2022) 55:9–23. doi: 10.1267/ahc.21-00081

4. Zhang X. Effect of metformin combined with clomiphene on insulin resistance , oxidative stress response and T cell immune response in patients with PCOS. *J Hainan Med Univ* (2017) 23:66–69.

5. Daneshjou D, Mehranjani MS, Zadehmodarres S, Shariatzadeh SMA, Mofarahe ZS. Sitagliptin/metformin improves the fertilization rate and embryo quality in polycystic ovary syndrome patients through increasing the expression of GDF9 and BMP15: A new alternative to metformin (a randomized trial). *J Reprod Immunol* (2022) 150:103499. doi: 10.1016/j.jri.2022.103499

6. Cheraghi E, Soleimani Mehranjani M, Shariatzadeh SMA, Nasr Esfahani MH, Alani B. N-acetylcysteine compared to metformin, improves the expression profile of growth differentiation factor-9 and receptor tyrosine kinase c-kit in the oocytes of patients with polycystic ovarian syndrome. *Int J Fertil Steril* (2018) 11:270–278. doi: 10.22074/ijfs.2018.5142

7. Iwata N, Hasegawa T, Fujita S, Nagao S, Nakano Y, Nada T, Nishiyama Y, Hosoya T, Otsuka F. Effect of the interaction of metformin and bone morphogenetic proteins on ovarian steroidogenesis by human granulosa cells. *Biochem Biophys Res Commun* (2018) 503:1422–1427. doi: 10.1016/j.bbrc.2018.07.058
